# Host Sex Steroids Interact With Virus Infection: New Insights Into Sex Disparity in Infectious Diseases

**DOI:** 10.3389/fmicb.2021.747347

**Published:** 2021-11-04

**Authors:** Jinfeng Wu, Lei Zhang, Xing Wang

**Affiliations:** Key Laboratory of Gastrointestinal Cancer (Ministry of Education), School of Basic Medical Sciences, Fujian Medical University, Fuzhou, China

**Keywords:** human viruses, virus infection, male predominance, androgen receptor, transmembrane protease/serine subfamily member 2

## Abstract

Sex hormones are steroid hormones synthesized from the gonads of animals and tissues such as the placenta and adrenocortical reticular zone. The physiological functions of sex hormones are complex. Sex hormones are not only pathologically correlated with many diseases of the reproductive system, but are etiological factors in some viral infectious diseases, including disease caused by infections of coronaviruses, herpesviruses, hepatitis viruses, and other kinds of human viruses, which either exhibit a male propensity in clinical practice, or crosstalk with androgen receptor (AR)-related pathways in viral pathogenesis. Due to the global pandemic of coronavirus disease 2019 (COVID-19), the role of androgen/AR in viral infectious disease is highlighted again, majorly representing by the recent advances of AR-responsive gene of transmembrane protease/serine subfamily member 2 (TMPRSS2), which proteolytically activates the receptor-mediated virus entry by many coronaviruses and influenza virus, along with the role of androgen-mediated signaling for the transcription of hepatitis B virus (HBV), and the role of sex hormone responsive genes during Zika virus (ZIKV) pathogenesis, et al. Collectively, we propose to provide a comprehensive overview of the role of male sex hormones during multiple phases in the life cycle of different human viruses, which may be partly responsible for the sex-specific prevalence, severity and mortality of some diseases, therefore, may provide clues to develop more efficient prevention and treatment strategies for high-risk populations.

## Introduction

The ongoing pandemic of COVID-19, caused by severe acute respiratory syndrome coronavirus 2 (SARS-CoV-2), highlights those viral infectious diseases still present a serious threat to human health (Guan et al., 2020; Rozenberg et al., 2020). This is also evident from the regional outbreaks of influenza (Iuliano et al., 2018), viral hepatitis (Lee and Banini, 2019; Megahed et al., 2020), Ebola virus disease (EVD) (WHO, 2016), and the Zika epidemic (Duffy et al., 2009; Musso et al., 2014), all of which exhibit a male propensity for virus infection and pathogenesis that is mediated by different mechanisms.

Male bias in COVID-19 mortality is observed in nearly all countries with available sex-disaggregated data, and the risk of death in males is 1.7 times higher than in females (Scully et al., 2020). This disparity was first reported in China, where, the death rate among men was 2.8% vs. 1.7% in women (Gemmati et al., 2020). Regarding the infection rate, the ratio in males to females was 3:1 in Italy (Gebhard et al., 2020). Notably, there are several other viruses, including Kaposi’s sarcoma-associated herpesvirus (KSHV), HBV, influenza virus, and Respiratory syncytial virus (RSV) which predominantly affect males (Tsay et al., 2009; Lorenzo et al., 2011; Jones et al., 2019; Orimadegun et al., 2020). KSHV, HBV, and hepatitis C virus (HCV) are three types of human oncoviruses, which are defined as sex hormone responsive (Bosch et al., 2004; Jemal et al., 2011; Bradley et al., 2020). It was reported that men are more susceptible to seasonal influenza virus, and that the clinical outcomes are more severe (Wong et al., 2019). Besides, invasion or damage of the male reproductive system are reported outcomes of viral infection with SARS-CoV-2, Ebola virus (EBOV), and ZIKV (Counotte et al., 2018; Den Boon et al., 2019; Vishvkarma and Rajender, 2020). Taken together, these data suggest the significant implications of sex hormones in the gender differences observed in virus-associated susceptibility, prevention, clinical manifestations, treatment, prognosis, and pathogenesis (Baggio et al., 2013).

Androgen receptor is a ligand-dependent nuclear transcription factor (Dai et al., 2017). It mainly works in combination with natural agonists such as testosterone and dihydrotestosterone (DHT). At the absence of the ligand, AR complex with heat shock proteins and immunophilin, anchoring to cyto-skeletal elements, and residing primarily in the cytoplasm (Smith and Toft, 2008). However, upon the binding of ligand, the homodimer of ligand-AR translocates from cytoplasm to nucleus and binds to the androgen response elements (AREs) of target genes, thus regulating the downstream transcription cascades (Claessens et al., 2001; Dai et al., 2017). TMPRSS2, a well-known AR-responsive gene, emerged as hot topics in COVID-19 by processing a universal proteolytical activating effect to coronavirus family and influenza virus (Kawase et al., 2012; Böttcher-Friebertshäuser et al., 2014; Yamamoto et al., 2016; Shen et al., 2017; McKee et al., 2020), thus emphasize the requirement to fully elucidate the molecular mechanisms which underling the sex disparity in several viral infectious diseases.

Most studies to date have investigated this discrepancy in terms of gender-specific immune responses, and the results have shown that females have a greater ability to elicit immune responses against infection (Moulton, 2018; Ortona et al., 2019). Some hypotheses, which are mainly discussed in the review, suggest a direct effect by male sex hormones on pathogen infection. TMPRSS2, which is expressed in an androgen-dependent manner, is utilized by SARS-CoV-2 for the priming of viral spike (S) protein, which is essential for viral entry into primary target cells and for viral spread in the infected host (Mjaess et al., 2020). Similarly, TMPRSS2 acts as the major hemagglutinin (HA)-activating protease of influenza A virus (IAV) in human airway cells and of influenza B virus (IBV) in type II pneumocytes (Limburg et al., 2019). To the early-stage infection of other viruses, AR is involved in the coordinated activation of Src/RSK1/EphA2 Ser897 signaling, which promotes primary infection by KSHV (Wang et al., 2017). On the contrary, 17β-estradiol (E2) can inhibit HCV spread and/or entry by activating G-protein-coupled estrogen receptor, GPR30, increasing matrix metalloproteinase 9 (MMP-9) activation and exporting to the extracellular space leading to cleavage of occludin in Domain D (Ulitzky et al., 2016). The well-documented role of HBV in hepatocellular carcinoma (HCC) indicates that a virus–host feedback loop between the X gene of HBV and AR is established in HBV-infected male hepatocytes (Zhu et al., 2011). Similarly, HCV core protein mediated the feedback loop between AR and JAK/STAT signaling pathway also plays a vital role in HCV infection (Kanda et al., 2008).

Hence, this review aimed to further analyze the literature concerning (i) the implication of the androgen/AR axis and its downstream signaling in primary virus infection, and (ii) other mechanisms mediated by sex steroids or associated molecules in the regulation of virus replication or viral pathogenesis. This review may provide strategies for endocrine-based interventions and personalized treatment for high-risk groups.

## Coronaviruses

**FIGURE 1:**
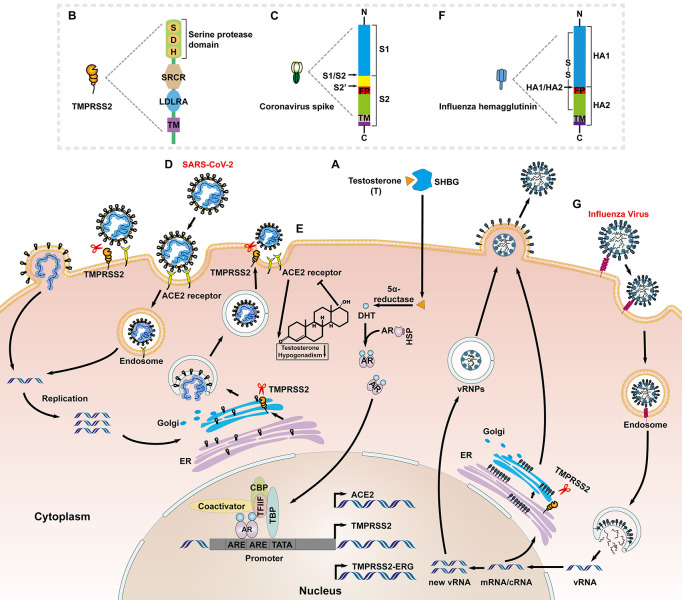
Priming of the SARS-CoV-2 S proteins and HA protein of influenza virus by TMPRSS2. **(A)** Sex hormone binding globulin (SHBG) transports androgens (mainly testosterone) and mediates cell entry. Intracellular 5α-reductases induce testosterone to produces active DHT that interacts with AR. The androgen and AR complex enters the nucleus and binds to ARE, then interacts with the TFIIF subunit, RNA polymerase II, TATA-binding polypeptide (TBP), CBP, AR co-activators, and other transcription factors to regulate the transcription of TMPRSS2. **(B)** The functional domains of TMPRSS2: the serine protease domain containing the catalytic triad consisting of histidine (H), aspartic acid (D) and serine (S); scavenger receptor cysteine-rich domain (SRCR); low-density lipoprotein receptor domain class A (LDLRA); and the N-terminal transmembrane domain. **(C)** The functional domains of coronavirus S protein: S1 subunit (attachment); and S2 subunit (fusion) including putative fusion peptide (FP) and transmembrane domain (TM); The S1/S2 and S2’ cleavage sites are indicated by black arrows. **(D)** ACE2 binds to the receptor-binding S protein of SARS-CoV-2 to accelerate the endocytosis of the virus into host cells; ‘TMPRSS2 cleaves the S2’ site of SARS-CoV-2 S protein to promote the direct fusion of viral particles to the cell and release of the viral genome, and activates S protein and cleaves the envelope protein on intracellular Golgi bodies to facilitate virus assembly and release. **(E)** A negative feedback regulation exist between the androgen/AR and ACE2. Lacking of androgen increases the expression of ACE2 in prostate cancer patients, and in turn ACE2 is constitutively expressed in adult-type Leydig cells which affects the secretion of testosterone. **(F)** The functional domains of influenza virus HA protein: HA1 subunit; and HA2 subunit including FP and TM; The HA1/HA2 cleavage site is indicated by a black arrow. **(G)** The HA1 subunit of the influenza virus HA protein binds to the sialic acid receptor to accelerate the endocytosis of virus particles into the cytoplasm. Influenza virus uses the released viral ribonucleoprotein complexes (vRNPs) as a template to transcribe and replicate, and then TMPRSS2 cleaves HA on intracellular Golgi bodies to facilitate virus assembly and release.

Transmembrane protease/serine subfamily member 2 is an androgen-regulated gene encoding a transmembrane serine protease (Figure 1A), which is located on human chromosome 21q22.3, and is approximately 66 kb in length (Shen et al., 2017). The full-length TMPRSS2 cDNA encodes a polypeptide of 492 amino acids, with a domain structure comprising a type II transmembrane domain, a receptor class A domain, a scavenger receptor cysteine-rich domain and a C-terminal ectodomain encompassing a large serine protease subunit (Figure 1B; Bottcher-Friebertshauser et al., 2010). It is well established that TMPRSS2 is highly expressed in prostate epithelial cells, and the TMPRSS2-ERG [erythroblast transformation-specific transcription factor (ETS) -related gene, ERG] fusion gene is frequently expressed in benign prostatic hyperplasia and primary prostate cancer tissues (Perner et al., 2007; FitzGerald et al., 2008; Park et al., 2014). In recent years, TMPRSS2 was confirmed to play a critical role in catalyzing virus–cell membrane fusion during SARS-CoV infection by proteolytically cleaving the S protein and activating its conformational flexibility (Heurich et al., 2014; Zmora et al., 2015). It is notable that this process is hijacked by a variety of human viruses, including middle east respiratory syndrome coronavirus (MERS-CoV) (Shirato et al., 2013), IAV (Tarnow et al., 2014; Cheng et al., 2015), and HCV (Esumi et al., 2015). Therefore, TMPRSS2 has emerged as a promising antiviral candidate in many types of infectious diseases (Kawase et al., 2012; Yamamoto et al., 2016; McKee et al., 2020).

Like other class I viral fusion proteins, the human coronavirus spike (S) proteins require proteolytic priming to be activated (Bertram et al., 2012; Zhou et al., 2015). Priming of coronavirus S by host cell proteases is essential for viral entry into cells (Figure 1). The S proteins include cell receptor-binding domains (RBDs) and virus–cell membrane fusion domains (Kirchdoerfer et al., 2016). The S1/S2 cleavage site of SARS-CoV-2 S harbors several arginine residues (multibasic residues), which indicate its high cleavability (Figure 1C). Hoffmann et al. (2020) showed that SARS-CoV-2 depends on furin-mediated precleavage of its S protein at the S1/S2 site for subsequent S protein activation by TMPRSS2 in lung cells (Figure 1D; Bestle et al., 2020; Hoffmann et al., 2020). Furin cleaves the R-R-A-R685↓ site in the S1/S2 domain of S protein, whereas, TMPRSS2 cleavage occurs at KR815↓ at the S2’ site (Coutard et al., 2020; Walls et al., 2020). For MERS-CoV, TMPRSS2 mediated precleavage at the S1/S2 motif (RSVR751), but this was not essential for subsequent virus activation. By contrast, the S2 site (RSAR) was required for efficient entry and the integrity of one of the two arginines was sufficient for cleavage, usually most of which are cleaved by TMPRSS2 or cathepsin L (Kleine-Weber et al., 2018). In the case of SARS virus, TMPRSS2 cleaved and activated the S protein at separate sites, i.e., R667 and R797 (Reinke et al., 2017).

Transmembrane protease/serine subfamily member 2 has been widely studied in the context of prostate cancer, where, it is highly expressed, and TMPRSS2 expression is increased in response to androgens through direct transcriptional regulation by the AR (Lin et al., 1999; Lucas et al., 2014). A 15-bp sequence of the androgen response element is located at position ∼148 from the putative transcriptional start site of TMPRSS2 (Figure 1A; Lin et al., 1999). This feature has been hypothesized to contribute to the high frequency of genomic rearrangements involving the TMPRSS2 promoter and ERG, which places this oncogene under AR control (Haffner et al., 2010). Taken together, inhibition of AR activity and downregulation of TMPRSS2 could be targeted to prevent SARS-CoV-2 infection (Stopsack et al., 2020). This hypothesis was partly supported by a retrospective study in Italy that found that prostate cancer patients receiving androgen deprivation therapy (ADT) were less susceptible to SARS-CoV-2 infection (Montopoli et al., 2020). The reasons for these gender disparities are still under investigation. Recent studies reported that the active genetic variant of AR, with a long CAG repeat, is associated with more severe COVID-19 disease (McCoy et al., 2021).

The cellular receptor of SARS-CoV-2, angiotensin-converting enzyme 2 (ACE2), was identified as another AR-regulated target (Majdic, 2020; Qiao et al., 2020; Figure 1A). Different from that of male sex hormones-activating TMPRSS2, a negative feedback regulation exists between the androgen/AR and ACE2 (Figure 1E). That is, ADT might increase ACE2 expression in patients of prostate cancer, which might be beneficial when SARS-CoV-2 competes with angiotensin II for binding sites (Cattrini et al., 2020). On the other hand, ACE2 is constitutively expressed in adult-type Leydig cells which affect the secretion of testosterone, and the mechanism may account for the testosterone deficiency in men infected with COVID-19 (Hackett and Kirby, 2020; Hussain et al., 2020; Giagulli et al., 2021). Transcriptional repression of the AR enhanceosome with AR or the bromodomain and extraterminal domain (BET) antagonists led to decreased expression of both TMPRSS2 and ACE2 in subsets of lung epithelial cells, and inhibited SARS-CoV-2 infection *in vitro* (Qiao et al., 2020). A few AR-binding motifs were also identified within ACE2 regulatory regions (Ragia and Manolopoulos, 2020), but it is still not known whether these elements actually cause the ACE2 promoter to exhibit an androgen-dependent response.

The full array of mechanisms responsible for the gender disparities observed in COVID-19 outcomes is likely to be multifactorial. The androgen/AR axis exhibits a multidimensional response to SARS-CoV-2 infection, from active genetic variants to transcriptional regulation of host entry factors by TMPRSS2 and ACE2.

## Herpesviruses

**FIGURE 2:**
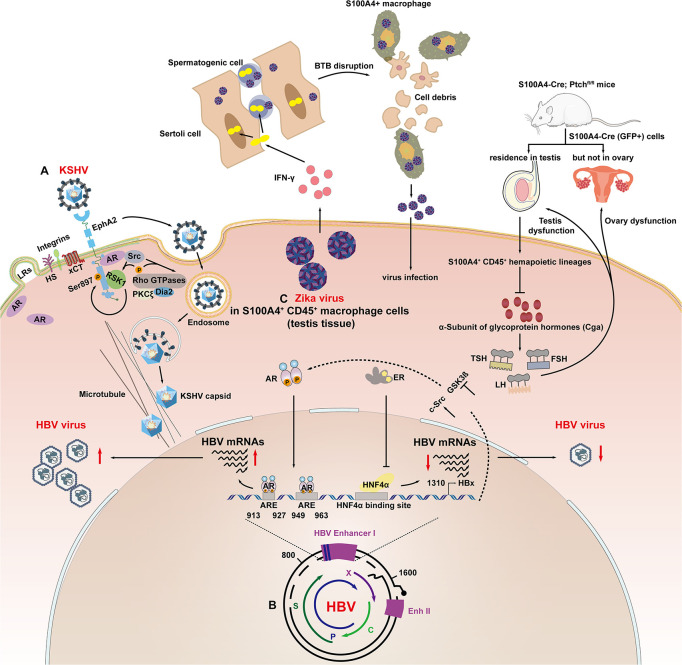
Sex hormone signaling axis modulates viral infection at the viral entry, transcription and replication stages. **(A)** AR mediated recruitment of Src, which led to the activation of the kinase p90 RSK1, which led to Ser897 phosphorylation of EphA2. AR coordinated activation of the Src/RSK1/EphA2 Ser897 signal to accelerate endocytosis of KSHV. **(B)** Androgen pathway increased the transcription of HBV mRNAs through direct binding to ARE1 (913–927 bp) and ARE2 (949–963 bp) in HBV enhancer I. The HBx gene can also enhance AR activity by activating c-Src signaling pathways and inactivating GSK-3β signaling pathways; ER-α binds to HNF-4α, causing molecular chelation, which inhibited the DNA binding ability of HNF-4α to HBV enhancer I, resulting in a decrease in enhancer I activity and HBV gene transcription. **(C)** ZIKV infection attracted S100A4 + macrophages to aggregate and differentiate into interferon-γ-expressing cells in the testis and secrete interferon-γ, increasing the permeability of the blood–testicular barrier (BTB), thereby facilitating the entry of ZIKV into the spermatogenic tubules; S100a4-Cre (GFP +) cells are expressed in the testis and rarely expressed in the interstitial tissue of the ovary. The interruption of the PTCH1 signaling pathway decreases the expression of Cga, which causes a decrease in the levels of follicular stimulating hormone (FSH), luteinizing hormone (LH), and thyroid stimulating hormone (TSH). Finally, the function of the ovaries and testes is damaged.

In contrast to the genomic regulation of AR to the key target genes, such as TMPRSS2 and ACE2, that promote coronavirus and influenza virus infection (Lin et al., 1999; Shen et al., 2017), membrane-localized AR has been shown for the first time to be associated with KSHV entry and endocytosis (Wang et al., 2017). Cell entry by KSHV is a multistep process involving viral envelope glycoproteins as well as several cellular attachment and entry factors (Chandran, 2010; Chakraborty et al., 2012). One such factor is ephrin receptor A2 (EphA2), which is localized to the cell membrane subdomains/lipid rafts, and Wang et al. (2017) demonstrated that AR act as a host factor to facilitate KSHV entry by mediating Src/RSK1/EphA2 Ser897 signaling cascades (Figure 2A). KSHV envelope glycoproteins H and L bind to EphA2 and trigger the phosphorylation of EphA2, thereby promoting endocytosis of KSHV, which involves the signaling cascades mentioned above (Akula et al., 2003; Raghu et al., 2009). The specific mechanism involves the AR-mediated recruitment of Src, leading to the activation of p90 ribosomal S6 kinase 1 (RSK1), which in turn, leads to Ser897 phosphorylation of EphA2 (Figure 2A). From these results, it can be concluded that the gender difference in KSHV infection may be related to the cascade of androgens that promote KSHV infection of endothelial cells and epithelial cells. This may also imply a new mechanism affected by gender differences in the pathogenesis of Kaposi’s sarcoma.

Zhang et al. (2018) recently reported that EphA2 is also a key receptor for Epstein-Barr virus (EBV) to infect epithelial cell. As a member of gamma-herpesvirus of human herpes virus type IV, the infection of EBV is also associated with sex hormones (Sasaki et al., 2020). Persistent EBV reactivation induces host B cells to differentiate into plasma cells and produce various isotypes of Ig, relating to autoimmune diseases (Hara et al., 2018), such as Graves’ disease (Hara et al., 2019) and Multiple sclerosis (MS) (Al-Temaimi et al., 2015), which occur predominantly in women. However, the progression of MS was more severe in male patients (55.1%) than in females (34.8%) (Al-Temaimi et al., 2015). In addition, studies have shown that the use of progesterone analog may decrease the DNA titer of EBV in plasma of cachectic patients with recurrent/metastatic nasopharyngeal carcinoma (NPC) (Hung et al., 2017). However, the underlying mechanism by which AR-pathways may involve in the process remains elusive and should be very interesting in the future work.

## Human Hepatitis Viruses

Virus-induced HCC is present worldwide, representing 60–80% of all liver cancer cases and the risk factors of viral infection with HBV is 54% of all HCCs, and/or HCV is 31% of all HCCs (El-Serag, 2012; Yang et al., 2019). Moreover, HBV and HCV infection showed explicit gender-related differences (Tsay et al., 2009; Bradley et al., 2020). However, the mechanisms of sex disparities in HBV and HCV infection are considerable differences.

Researchers reported that the rate of spontaneous clearance of HBV/HCV was higher among HBV/HCV-infected females when compared with male patients (Bakr et al., 2006; Wang et al., 2007), and sustained virologic response (SVR) was more prominent among male patients (Akuta et al., 2007; Iyer et al., 2017). Differences in immune capacity between men and women may be part of reasons to this bias, and it was reported that number of monocytes, macrophages and dendritic cells, which participated in preventing human from virus infection were higher in females (Klein, 2012). Besides, women usually produce more effective immune responses than men after virus infection (Ruggieri et al., 2016). On the other hand, gender bias in virus entry, transcription and replication can also contribute to this bias.

Sodium taurocholate co-transporting polypeptide (NTCP) has been supposed to play a key role for HBV entering (Yan et al., 2012), and it was reported that NTCP mRNA and protein expression were higher in male than female in rat hepatic sinusoid (Simon et al., 2004). Similarly, the entry of HCV for human liver cells also display gender differences. Scavenger receptors, which are critical for HCV entry were reported could be up-regulated by testosterone whiling be down-regulated by estrogen (Langer et al., 2002; Scarselli et al., 2002; Stangl et al., 2002; Evans et al., 2007; Meng et al., 2011). In addition, E2 was also reported to inhibit HCV entry through down-regulation of occludin (OCLN), which is also critical for viral infection (Ulitzky et al., 2016), it can activate GPR30, increase MMP-9 activation and export to the extracellular space leading to cleavage of occludin in Domain D, disrupting occludin-occludin and occludin-claudin-1 interaction.

Sex hormone pathways classically modulate HBV infection by activating their cognitive nuclear receptors, which act as transcriptional regulators, controlling the expression of HBV genes (Figure 2B; Wang et al., 2009, 2012). Wang et al. (2009) reported that the androgen pathway can increase the transcription of HBV mRNAs through direct binding to ARE1 (913–927 bp) and ARE2 (949–963 bp) in HBV enhancer I (Ruggieri et al., 2018). In turns, studies reported that HBx could promote AR transcriptional activity through glycogen synthase kinase-3β (GSK-3β) and c-Src kinase pathway (Chiu et al., 2007; Yang et al., 2009). This forms a positive loop which promotes the progression of HBV infection seriously in males. On the contrary, Wang et al., also concluded that estrogen receptor-α (ER-α) could reduce transcription of the HBV gene by suppressing HBV enhancer I activity. The hinge region and the boundary region between the DNA binding domain (DBD) and the hinge (amino acids 252–263) of ER-α bind to hepatocyte nuclear factor-4 alpha (HNF-4α), causing molecular chelation, which inhibited the DNA binding ability of HNF-4α (1134–1146 bp) to HBV enhancer I, resulting in a decrease in enhancer I activity and HBV gene transcription (Wang et al., 2012, 2015).

The sex hormone signaling axis is also associated with HCV replication, E2 was found to inhibit production of mature HCV virions at the virion assembly/secretion phase through binding to ER-α (Hayashida et al., 2010) according to Magri et al. (2017) estrogen pathway inhibited hepatitis C virus acting by interfering with assembly/release phases of its life cycle. In addition, one study suggested selective estrogen receptor modulators (SERMs) seemed to target multiple steps of HCV viral life cycle such as replication and post replication events, and it may be potential candidates for the treatment of HCV infection (Murakami et al., 2013). Interestingly, the HCV virus production also affects the sex hormone signal axis. Kanda et al. (2008) have demonstrated that HCV core protein can enhance AR-mediated transcriptional activity by activating JAK/STAT signaling pathway. These aforementioned studies might elucidate the role of sex hormone in modulating HCV infecting hepatocytes cells which may contribute to the sex bias in HCV infection.

## Other Viruses

Besides coronaviruses family, TMPRSS2 function in a similar way to the *de novo* infection of IAV, which is also regulated by androgen/AR axis (Figure 1A). Bottcher-Friebertshauser et al. (2010) first suggested that TMPRSS2 and human airway trypsin-like protease (HAT) are candidates for proteolytic activation of influenza viruses *in vivo* (Bottcher-Friebertshauser et al., 2010). HA was a prerequisite for successful binding to sialic acid-containing cell surface receptors and fusion between viral and endosomal membranes during virus entry. Cleavage of HA is essential for infection and determines viral pathogenicity and tissue tropism (Sakai et al., 2016). HA is synthesized as a precursor protein, HA0, and needs to be cleaved by a host cell protease into subunits HA1 and HA2 to gain fusion capacity (Figure 1F; Bottcher-Friebertshauser et al., 2010; Sakai et al., 2016). The residues of HA comprise the N-terminal of the scissile bond (↓) P1 and the C-terminal P1’; corresponding residues in the substrate binding domain of the activating enzyme are designated S1 and S1’ (Schechter and Berger, 1967; Luczo et al., 2015). Proteolytic cleavage of HA has been demonstrated to occur on the smooth membranes within the trans-Golgi network or at the cell surface for highly pathogenic and lowly pathogenic viruses, respectively (Bottcher-Friebertshauser et al., 2010). Unlike the membrane priming of TMPRSS2 to the S protein of SARS-CoV-2, membrane-bound TMPRSS2 cleaved the HA but this did not necessarily lead to its proteolytic activation (Böttcher-Friebertshäuser et al., 2014). Meanwhile, the newly synthesized HA within the cell was also cleaved by TMPRSS2, most probably during the transport of HA from the endoplasmic reticulum to the plasma membrane, where, virus assembly and budding take place (Figure 1G; Bottcher-Friebertshauser et al., 2010; Böttcher-Friebertshäuser et al., 2014). HAT and TMPRSS2 mediate proteolytic cleavage at a highly conserved arginine residue (Baron et al., 2013; Garten et al., 2015). Cleavage activation of TMPRSS2 was shown to occur autocatalytically. TMPRSS2 seems to possess only marginal enzymatic activity at the cell surface, whereas, HAT is a fully enzymatically active protease at the cell surface (Garten et al., 2015). These results further support the concept that TMPRSS2 cleaves the viral glycoproteins in different cellular compartments, leading to different functionality. Taken together, these findings indicate that potent protease inhibitors targeting TMPRSS2 are potential novel drugs for virus treatment.

Zika virus, a neglected mosquito-borne *Flavivirus*, was recently reported to establish long-term infection in the testes by preferentially infecting spermatogonia, primary spermatocytes and sertoli cells, thus resulting in sexual transmission and impaired male fertility (Lozier et al., 2018; Clancy et al., 2019). Our understanding of the mechanisms involved was recently advanced, as shown in Figure 2C. Yang et al. (2020) reported that S100A4 + macrophages may facilitate ZIKV crossing of the blood–testis barrier in multiple ways, establishing a biological association between male steroids and ZIKV infection.

Both the calcium binding protein S100A4/Mts1 and its endogenous receptor (receptor for advanced glycosylation end products; RAGE) have been implicated in the development of sex hormone-dependent formation of the cortical bone (Erlandsson et al., 2013) and pulmonary arterial hypertension (Dempsie et al., 2011). It was shown that physiological concentrations of E2 increased S100A4 expression led to cell proliferation, which was inhibited by soluble RAGE, by antagonizing the membrane-bound form of RAGE. Estrogen-related receptor γ (ERRγ) promotes the aggressiveness of endometrial cancer by activating the transcription of S100A4 (Hua et al., 2018). In feedback regulation, Ren et al. (2019) reported that the activity of the S100A4 promoter-driven Cre recombinase (S100A4-Cre) is restricted to CD45 + cells of hematopoietic origin, causing sex-specific changes in the expression of genes in regulating fertility and endocrine function. Consistent with this, the CD45 + myeloid macrophage subpopulation located in mice testes, which is susceptible to ZIKV infection, comprised mainly of S100A4 + cells. Mechanically, it was demonstrated that interferon-γ secreted by S100A4 + macrophages induced the tight junction protein Claudin-1 to translocate from the plasma membrane into the nuclei, thus increasing the permeability of the blood–testis barrier (an indispensable structure surrounding the seminiferous tubules and protecting the spermatogenic cells inside from viral infection and immune attack). Whether S100A4 directly functions in ZIKV infection through a sexually dimorphic mechanism remains to be determined. A few studies have also shown the various roles of S100A4 + cells in the pathogenesis of sexually transmitted viruses, which indicates that S100A4 is a promising target of viral infectious diseases.

The infection of RSV is also showed a male propensity in severe RSV bronchiolitis. Meta-analyses showed that the gender of male is a known risk factor for the disease (Lanari et al., 2015; De Jacobis et al., 2020; Orimadegun et al., 2020). A recent study showed that upon androgen treatment, higher amounts of RSV were detected in body fluids in comparison to solvent (Echchgadda et al., 2011). The current researches contribute the sex difference usually from the aspects from the immune discrepancy between genders (Carvajal et al., 2019), the possible direct mechanisms by which AR axis may crosstalk with the virus are lacking.

Finally, the outbreaks of EVD are clinically evidenced a sexual transmission. Even after 6 months to more than one year after recovery from EVD, male patients can also transmit the EBOV to their partner (Mate et al., 2015). And the EBOV RNA can be detected from semen tissues up to 965 days, from the onset of EVD (Fischer et al., 2017). Therefore, the research into the sexual transmission mechanism of ZIKV may provide a reference for studying the disparity in related diseases caused by EBV infection.

## Conclusion

Currently, our knowledge on the potential mechanisms determining sex disparity in infectious diseases, either immunological or via signaling pathways, is fragmented and not exhaustive. The male predominance of multiple types of human viruses has been clinically observed in viral infectious diseases; however the underlying mechanisms of male sex steroids action need further investigation, especially its versatile functions by targeting on different stages of virus lifecycle. In terms of the initial virus infection, the infection pathway is likely to influence the duration and severity of infection. Thus, in-depth studies on the function of sex hormones in each specific step of the virus life cycle will be vital. The molecular mechanisms responsible for sex bias in virus infections are only beginning to be revealed; however, identification of the key molecules and signal pathways involved may provide new insight into identifying high-risk groups and disclosing new targets for personalized medicine, targeted drugs and vaccinology.

## Author Contributions

JFW contributed to the conception, design, and writing of the manuscript. LZ contributed to manuscript drafting of the revised one. XW contributed to the conception, writing, and critical reviewing of the manuscript. All authors made the final approval and the agreement to account for all aspects of the manuscript.

## Conflict of Interest

The authors declare that the research was conducted in the absence of any commercial or financial relationships that could be construed as a potential conflict of interest.

## Publisher’s Note

All claims expressed in this article are solely those of the authors and do not necessarily represent those of their affiliated organizations, or those of the publisher, the editors and the reviewers. Any product that may be evaluated in this article, or claim that may be made by its manufacturer, is not guaranteed or endorsed by the publisher.
